# The History and Philosophy of Ecological Psychology

**DOI:** 10.3389/fpsyg.2018.02228

**Published:** 2018-11-27

**Authors:** Lorena Lobo, Manuel Heras-Escribano, David Travieso

**Affiliations:** ^1^Facultad de Ciencias de la Salud y de la Educación, Universidad a Distancia de Madrid, Madrid, Spain; ^2^Department of Logic and Philosophy of Science, IAS-Research Centre for Life, Mind and Society, Universidad del País Vasco-Euskal Herriko Unibertsitatea, San Sebastian, Spain; ^3^Facultad de Psicología, Universidad Autónoma de Madrid, Madrid, Spain

**Keywords:** ecological psychology, Gibson, perception-action, affordances, specificity, perceptual learning, pragmatism

## Abstract

Ecological Psychology is an embodied, situated, and non-representational approach pioneered by J. J. Gibson and E. J. Gibson. This theory aims to offer a third way beyond cognitivism and behaviorism for understanding cognition. The theory started with the rejection of the premise of the poverty of the stimulus, the physicalist conception of the stimulus, and the passive character of the perceiver of mainstream theories of perception. On the contrary, the main principles of ecological psychology are the continuity of perception and action, the organism-environment system as unit of analysis, the study of affordances as the objects of perception, combined with an emphasis on perceptual learning and development. In this paper, first, we analyze the philosophical and psychological influences of ecological psychology: pragmatism, behaviorism, phenomenology, and Gestalt psychology. Second, we summarize the main concepts of the approach and their historical development following the academic biographies of the proponents. Finally, we highlight the most significant developments of this psychological tradition. We conclude that ecological psychology is one of the most innovative approaches in the psychological field, as it is reflected in its current influence in the contemporary embodied and situated cognitive sciences, where the notion of affordance and the work of E. J. Gibson and J. J. Gibson is considered as a historical antecedent.

## Introduction

This article has been written to be part of the research topic “History of Psychology as a Scientific Discipline,” an article collection for Frontiers in Psychology. Here we offer a succinct introduction to the history and philosophy of ecological psychology for the general reader, aiming to complement former introductions and works offered by researchers within this approach.

Ecological psychology is an embodied, situated, and non-representationalist approach to cognition pioneered by J. J. Gibson (1904–1979) in the field of perception and by E. J. Gibson (1910–2002) in the field of developmental psychology. Ecological psychology, in its very origins, aimed to offer an innovative perspective for understanding perception and perceptual learning that overcomes the traditional psychological dichotomies of perception/action, organism/environment, subjective/objective, and mind/body. These dichotomies are at the basis of some theoretical assumptions in the field of psychology, such as the poverty of stimulus and the passivity of perception. Precisely, the ecological approach challenged these widely accepted ideas that mainstream experimental psychology sustained during its run-up at the first half of the 20th century. Thus, once the framework of ecological psychology was established during the second half of the 20th century, it became an alternative in the debate between cognitivism and behaviorism. While both approaches considered themselves as competitors, they were taken as complementary from an ecological standpoint (Reed, 1991). This is so because they emphasized distinct stages of a whole cognitive picture sustained by the same principles. The ecological approach rejected the inferential and representational commitment of cognitivism and the physicalist idea of stimulus of behaviorism.

The main aim of this article is to offer a systematic introduction to the history and theory of ecological psychology, highlighting the academic biographies of the main proponents of this framework and the philosophical concerns that guided its genesis and development. It is not our objective to give an exhaustive account of the framework, but to offer a comprehensive overview of its main assumptions and achievements. For doing this, in section Theoretical Influences and the Genesis of Ecological Psychology we analyze the theoretical influences of J. J. Gibson and E. J. Gibson that shaped the ecological approach to perception from the 1920s to the 1950s. We focus on pragmatism, behaviorism, phenomenology, and Gestalt theory as main influences. In section The Establishment of the Ecological Account, we sketch the main principles of ecological psychology that originated from the 1960s to the 1970s and their reception in the neogibsonian literature. These principles are perception-action, perceptual systems, organism-environment system, ecological information, specificity, affordances, and perceptual learning. In section Relevant Developments, we explain the main progresses of the ecological approach from the 1980s to the 2010s, emphasizing the role of visual control of action and Lee’s tau (τ) theory and its influence in the ecological approach. At this point, we also highlight the developments of the so-called Connecticut School (Heft and Richardson, 2013) and other relevant studies included in the neogibsonian framework.

## Theoretical Influences and the Genesis of Ecological Psychology

In this section we analyze the main influences that gave rise to ecological psychology. We identify three main sources of influence: James’ radical empiricism and neutral monism, behaviorism, phenomenology, and Gestalt theory.

### Pragmatism

The first source of inspiration^1^ that J. J. Gibson took into account for developing the ecological approach to cognition was American pragmatism and, in particular, the ideas of James on radical empiricism and neutral monism. J. J. Gibson was highly influenced by Holt, who taught him the main principles of radical empiricism at Princeton, were J. J. Gibson got his B.Sc., M.A., and Ph.D. (1925, 1926, and 1928, respectively) (Hochberg, 1994, pp. 152–153).

According to pragmatism, practical consequences should be taken as more relevant than abstract principles to explain scientific practices, ethics, and cognition. This school rejects some shared aspects of idealism and empiricism (the passivity of perception and the representationalist account of the mind) and focuses on the active capacities of organisms and their adaptation to the environment for explaining cognition. According to pragmatism, “individuals can never know the world independently of their own experience” (Heft, 2001, p. 74). James’ version of pragmatism includes an epistemic thesis, radical empiricism, and a metaphysical thesis, neutral monism. These two theses are intertwined. Radical empiricism claims that our knowledge comes from experience, which is taken as the capacity of engaging into meaningful interactions with the world. These meaningful interactions include objects of perception, the relations among those objects, and also the relations of those objects to us. Thus, we experience those relations in a meaningful and organized way. James concludes that we can describe this relational world of pure experience either from the side of the object (the sense datum) or from the side of the subject (the experience). Therefore, this approach is called neutral monism: there is only one stuff that can be described physically or psychologically (James, 1895, p. 110).

The influence of radical empiricism in J. J. Gibson’s view is quite clear: he claimed in his autobiography that he was a radical empiricist J. J. Gibson (1967) and this view is at the very basis of his description of affordances. Having in view the Jamesian idea that pure experience can be described either physically or psychologically, he defined affordances as “both physical and psychical, yet neither” (J. J. Gibson, 1979/2015, p. 121). This description of affordances, as we can see, cannot be understood without appealing to the influence of radical empiricism and neutral monism.

In his last year as undergraduate student at Princeton, J. J. Gibson took a course with Langfeld in experimental psychology. The year after, Langfeld offered him an assistantship. That year, Holt moved to Princeton, and taught J. J. Gibson the principles of behaviorism and radical empiricism. Since then, in J. J. Gibson’s words: “[f]or 30 years I was reluctant to abandon it [Holt’s motor theory of consciousness]” (J. J. Gibson, 1967, p. 129). For all these reasons, historians of ecological psychology understand that J. J. Gibson’s approach to perception is an experimental version of James’ radical empiricism (Heft, 2001).

### Behaviorism

Many authors understand ecological psychology as a version of behaviorism (Costall and Morris, 2015) and it is true that, in his own words, J. J. Gibson “became excited by the behaviorist revolution” (J. J. Gibson, 1967, p. 128). We think that the origin of the sympathies of J. J. Gibson toward behaviorism came from Holt’s unorthodox understanding of behaviorism.

Although Holt was close to James and some of his ideas, he did consider himself neither a pragmatist nor a behaviorist (Langfeld, 1946, pp. 251–255). He endorsed a Jamesian concept of consciousness; this is, he understood consciousness as an activity rather than as a reified object of study. But, at the same time, he combined this idea with the methods of behaviorism (although reacting against the passivity of perception usually accepted by this approach), creating his own motor theory of consciousness. Holt also included in his explanations aspects of behavior rejected by traditional behaviorism, such as goals or plans (Holt, 1914). In fact, the problem of purposive behavior is much more emphasized in Holt’s explanation of behavior than in traditional behaviorism.

At his respect, note that Tolman, another student of Holt, emphasized purposive behavior in his behaviorist view and developed the idea of ‘sign Gestalt’ as external relations among objects that explain their demanding character (see section Gestalt Psychology). This idea is quite similar to J. J. Gibson’s idea of affordance (Warren, 1984, p. 684). E. J. Gibson also found similarities between J. J. Gibson’s notion of affordance and Tolman’s idea of ‘manipulanda’ for explaining what he called behavior-support. According to Tolman, behavior-support were characters in the environment that helped behavior-acts to go without disruption. Manipulanda were the characters of objects that supported motor activity (properties such as lengths, fluidities, or solidities) that were not defined ‘in themselves’ but with regard to the kinds of manipulations that they support for a given organism (sit-in-able-nesses, pick-up-able-nesses, etc.) (Tolman, 1932, p. 448, as cited in E. J. Gibson, 1982, p. 61). Although there are key differences between J. J. Gibson’s ecological approach and Tolman’s behaviorism, there are enough similarities between manipulanda and affordances to consider the former as an inspiration of the latter.

It is also worth mentioning that E. J. Gibson was fond of this sort of behaviorism with a pragmatist turn. Her work was highly influenced during her Ph.D. studies by Hull (1929) another behaviorist. In particular, his paper “A functionalist interpretation of the conditioned reflex”, where he reinterpreted the behaviorist conditioned reflex in a pragmatist way, was specially inspiring for her (E. J. Gibson, 1991, 2002; p. 5, 28).

Despite its influence, ecological psychology offers some of the most powerful arguments against behaviorism. J. J. Gibson rejected the behaviorist idea of stimulus for two reasons: first, because it was measured as a physical unit (light, pressure, waves of sound) unrelated to the agent’s capacities; second, because it was passively received by the senses, whereas ecological psychology emphasized the active exploratory role of the agent. Therefore, J. J. Gibson understood the senses as perceptual systems (J. J. Gibson, 1966), as we will see in section Perception-Action Loop and Perceptual Systems. The behaviorist idea of stimulus was subsumed within the cognitivist approach, where it was enriched and stored to generate representations (Reed, 1991; see also section Perceptual Learning), something that J. J. Gibson and E. J. Gibson (1955) directly rejected. Thus, he developed his own idea of ‘stimulus information,’ now named ecological information that we analyze in section Organism-Environment System, Ecological Information, and Specificity.

### Gestalt Psychology

Koffka, Wertheimer, and Köhler were the main representatives of Gestalt psychology, a theory that defended a phenomenological approach to perceptual consciousness understood as the primacy of the mental organization. Gestaltists embraced some kind of dualism between the mental and physical realms, and suggested that mental organization mimics, or is isomorphic to, physical properties.

Gestalt theory influenced ecological psychology in many ways. First, as shown in the previous section, the varieties of pragmatism and behaviorism in which J. J. Gibson was educated prevented him from accepting the anti-positivist and anti-empiricist theses of Gestalt psychology. In his doctoral dissertation, J. J. Gibson refuted the explanation of Wulf, a student of Koffka in Berlin, who argued that memories of visually perceived forms are reproduced according to the principles of Gestalt organization. J. J. Gibson (1929) published these results in the *Journal of Experimental Psychology* in 1929 and interpreted them as showing the effect of perceptual habits according to Holt’s quasi-behaviorism. In J. J. Gibson’s words:

“The types of change here observed may all be explained, it is believed, by the supposition that the experience of the individual has brought into existence certain habitual modes of perception, and that these perceptual habits, rather than the laws of configurations, condition the changes observed” (J. J. Gibson, 1929, p. 35).

On the other hand, J. J. Gibson confronted the elementarist-inferential tradition of experimental psychology by arguing against the sensation-perception dichotomy and the poverty of stimulus. He did so by appealing to the ideas of higher order variables (like optic flow, see sections Organism-Environment System, Ecological Information, and Specificity and Visual Control of Action and Tau Theory) and meaning thanks to the notion of affordances (possibilities for action, see section Affordances). Some authors interpreted his arguments and ideas as inspired by the Gestalt tradition (e.g., Jenkins, 2008; Richards, 2012).

After obtaining his Ph.D. in Psychology in 1928, J. J. Gibson moved to teach Psychology at Smith College, where he became a colleague of Koffka. They were in contact until 1941, when Koffka passed away and J. J. Gibson joined the Air Force. An essential aspect that J. J. Gibson inherited from Gestalt psychology is the idea that the objects of perception (the Gestalten) are our primary way of engaging with the world, that the experience is given to us by certain laws that shape it, and that there is no point in reducing those objects into simpler physical units or elements that are recombined in our head because they are already structured and meaningful. Although J. J. Gibson never accepted the Gestaltist distinction of the mental and the physical, the ideas of the irreducible aspect of our experience and the seek for its lawful description were taken by J. J. Gibson and lead him to develop the concepts of affordance and ecological scale (the psychological level for explaining behavior, see section Organism-Environment System, Ecological Information and Specificity).

Regarding affordances, J. J. Gibson accepted the Gestaltist conception of the stimulus as having a functional relation with the perceiver. Gestaltists understood that meaning or values were subjectively imposed to the natural word, and they offered different characterizations for explaining this. Lewin developed the word Aufforderungscharakter, while Koffka preferred the term ‘demanding character’ (J. J. Gibson, 1979/2015; p. 130; E. J. Gibson, 1982, p. 61). However, J. J. Gibson (1982, p. 409) rejected an essential aspect of this idea: “[t]he crux of this theory is that the demand character, like the valence, was assumed to be in the *phenomenal* but not in the physical object.” He believed that this character was not subjectively imposed.

### Phenomenology

Another tradition that has been influential for the development of J. J. Gibson’s approach is phenomenology (Heft, 2001, pp. 114–123). J. J. Gibson would have learned about phenomenology thanks to Langfeld, chairman of the psychology department at Princeton, who in turn studied in Berlin in 1903 under Carl Stumpf, an early advocate of phenomenology and student of Franz Brentano; also, J. J. Gibson was colleague of Fritz Heider and Kurt Koffka at Smith College, and both helped bring the phenomenological approach from Europe to the United States (Heft, 2001, p. 117). Phenomenology is a philosophical approach that aims to explain the structures that allow us to have experience of the world (Käufer and Chemero, 2015); or, in other words, to offer “a direct description of our experience as it is without taking account of its psychological origin and the causal explanations which the scientist, the or the sociologist may be able to provide” (Merleau-Ponty, 1945/2012, p. vii). Although J. J. Gibson developed a scientific approach, the idea of offering a non-reductive “direct description of our experience” goes in line with his views.

In particular, J. J. Gibson’s approach has been said to parallel the work of Merleau-Ponty (Heft, 2001, p. 93, footnote 17, p. 117; Glotzbach and Heft, 1982). Beyond the previously mentioned historical account, authors such as Mace (2014) claimed that J. J. Gibson wrote some notes in the early 1970s on Merleau-Ponty (1945/2012) *Phenomenology of Perception* (Chemero and Käufer, 2016, p. 67). According to those notes, Merleau-Ponty exerted considerable influence on Gibson, especially regarding the ideas of occlusion and depth.

However, besides those ideas, and after a careful reading of each author’s works, there is reason to believe that Merleau-Ponty’s notion of body schema was also influential for J. J. Gibson. Merleau-Ponty’s approach to phenomenology was quite unique at the time: he believed that the structures that allowed for cognition were not cultural or purely mental, but bodily structures. Perceiving is the primordial way of knowing, but in this view, perception is the result of *comporting* toward the surrounding objects in a *meaningful* sense (Merleau-Ponty, 1945/2012). Thus, in this view, dealing with the environment is an exploratory activity of the agent that is also guided by the environment, something quite similar to the action-perception loop of the organism in ecological psychology. This gives rise to the idea of the body schema, a central concept in Merleau-Ponty’s phenomenology. It is the pre-conscious system that emerges by the combination of the bodily capacities of the agent and the complementing aspects of the environment (Toadvine, 2016). As defined by Merleau-Ponty, “[t]he body schema is, in the end, a manner of expressing that my body is in and toward the world” (Merleau-Ponty, 1945/2012, p. 103). Note the similarities with the idea of organism-environment mutuality: in both pictures there is a reciprocity of actions of the agent and elements of the environment that together make emerge a meaningful history of interactions. This Merleau-Ponty an configuration of our surrounding elements as connected to our capacities and actions is what provides experience with meaning (Kelly, 2004), and this is in line with the idea of the ecological meaning of the environment for the agent. The exploratory capacities of the agent are of course active, “causing a thousand signs to appear there, as if by magic, guide action” (Merleau-Ponty, 1945/2012, p. 115). The environment also guides and constraints behavior in this approach through signs, and those signs are also partially generated by the active capacities of agents. This is clearly in line with the Gibsonian view by which the combination of action and environmental elements gives rise to the emergence of ecological information and affordances. Those signs are similar to affordances, because they are meaningful in an embodied way and guide the agent’s behavior. Besides, this body schema of actions and signs generates a network of attractions and repulsions similar to the way in which J. J. Gibson introduced the idea of positive and negative affordances. As we can see, there are many elements in the Gibsonian account that parallel the ideas of Merleau-Ponty.

## The Establishment of the Ecological Account

The birth of the ecological account can be followed through the work of the Gibsons and their collaborators. J. J. Gibson’s joined the Air Force in the 1940s and his work on aviation studies lead him to publish *The perception of the visual world* (1950) (Lombardo, 1987/2017). In this book, J. J. Gibson took the retinal image as the basic visual stimulus for perception, although his concept was not equivalent to a static picture, as it was conceived at that time. In the next decade, J. J. Gibson substituted the idea of retinal image with the optic array (Lombardo, 1987/2017) (see section Organism-Environment System, Ecological Information, and Specificity). In the 1960s, he published *The senses considered as perceptual systems*, a book in which the traditional Aristotelian account of the senses changed to be defined as active “systems rather than channels” (J. J. Gibson, 1966, p. 47). In the same decade E. J. Gibson published *Principles of perceptual learning and development* (1969), where she proposed a working principle related to the concept of specificity: the principle of reduction of uncertainty. Her work delved into ontogeny and perceptual learning (as we will see in section Perceptual Learning) and on reading (E. J. Gibson and Levin, 1975), while J. J. Gibson focused on perception. He published his most famous book, *The ecological approach to visual perception* (J. J. Gibson, 1979/2015), only a few months before his death. E. J. Gibson survived her husband for more than 20 years, publishing two more scientific books (E. J. Gibson, 1991; E. J. Gibson and Pick, 2000) and one autobiography (E. J. Gibson, 2002), among other things.

In this section, we have selected the main ideas that guided the development of this psychological tradition and its reception in the neogibsonian framework.

### Perception-Action Loop and Perceptual Systems

The relationship between perception and action has been a major topic in the psychological literature before the birth of the ecological account. For example, Dewey (1896) argued against explaining behavior through sensory stimulus and motor responses. He proposed the sensorimotor coordination process for explaining experience. The ecological tradition agrees with Dewey’s idea that perception must be considered an active process in which the agents’ exploration, perception, and action are parts of the same activity. Although J. J. Gibson did not mention Dewey as an influence, E. J. Gibson cited Dewey and presented his work as an antecedent of the ecological approach (E. J. Gibson, 1988, p. 5):

“We don’t simply see, we look. The visual system is a motor system as well as a sensory one. When we seek information in an optic array, the head turns, the eyes tum to fixate, the lens accommodates to focus, and spectacles may be applied and even adjusted by head position for far or near looking. This is a point long emphasized by functional psychologists such as Dewey (1896) and Woodworth (1958). It was developed in detail by Gibson – e.g., in his experiments on active touch (1962).”

One of the first works of J. J. Gibson that conceived perception as an active process is found in the experimental studies on driving that he performed during the 1930s. J. J. Gibson and Crooks (1938) realized that the analysis of driving behavior could be made as a variation of Lewin (1936) ideas on locomotion (1936) introducing dynamic concepts such as the visual field, a precursor of optic flow (see section Visual Control of Action and Tau Theory). On the one hand, this study is interesting because it is another example of the influence of Gestalt theory in J. J. Gibson. On the other hand, this early work considers perception beyond the static, contemplative process traditionally depicted during the 19th century. The active character of perception was fully accepted for J. J. Gibson in subsequent years and, finally, perception and action were presented as interdependent in J. J. Gibson (1966) book. In this book, he emphasized the exploratory activity, including head and eye movements for vision and hand movements for haptics, for example. Consistently, J. J. Gibson’s idea of perceptual systems implied that there is an intrinsic coordination of perception and action when one of these systems is functioning. Following J. J. Gibson (1966) contribution, sense modalities should not be understood as subpersonal systems in which specialized receptors (photoreceptors, for example) are stimulated by a sensory stimulus (photons) producing a sensory impression that is enriched or transformed to form a representation. According to J. J. Gibson, animals evolved not only to be sensitive to sensory stimuli, but also to detect ecological information. What is needed for this is not only the subpersonal neural pathways and systems, but also a range of behaviors that are instrumental in revealing and picking-up ecological information: these behaviors extend to the eye-brain connection and include movements in the eyes, head, and body that facilitate the detection of ecological information in the array. Thus, a sense modality is considered as a perceptual system when it includes this range of behaviors of the animal taken as a whole (Glotzbach and Heft, 1982, p. 112). One of the most interesting topics in this book is the haptic system, especially *dynamic touching* (1966, p. 127), the term that he proposed for the active touch that involves exploration, wielding, and manipulation of objects. It is also interesting to note that J. J. Gibson also advanced the importance of inertia for dynamic touch, a research field that still has a very important research agenda, as we will see in section Dynamic Touch.

Regarding the concept of perception-action loop, the ecological approach includes the active character of perception as one of its distinctive features. Richardson et al. (2008, p. 174) clearly summarized the ecological viewpoint remarking that the perception-action cycle is not a merely reformulation of the interaction or influence of two processes, but the statement that “perception and action are of the same logical kind, and are mutual, reciprocal, and symmetrically constraining.” Thus, perceptual systems orient the perceptual organs and make adjustments in the exploration to resonate when ecological information is picked up. Its picking up is the reason why organism and environment are entangled in action-perception dynamics (see Warren, 2006, for a comprehensive explanation of perception-action loops from a neogibsonian framework).

### Organism-Environment System, Ecological Information, and Specificity

The organism-environment duality is probably the most important dichotomy that ecological psychology aimed to overcome. This traditional dichotomy has a broader scope than the mind-body problem, which has been extensively discussed in psychology (Michaels and Carello, 1981), and its implications are even more relevant that the ones produced by the rejection of the perception-action dichotomy that we outlined in section Perception-Action Loop and Perceptual Systems. As Costall (2004) pointed out, the pragmatist tradition and its relation to Darwinian ideas directly influenced ecological psychology for rejecting the organism-environment dualism. If one follows the academic biography of J. J. Gibson, organism-environment mutuality is not present in his early work on psychophysics (Lombardo, 1987/2017). However, his thought evolved to recognize an essential reciprocity between perceiver and environment, as he clearly stated it at the beginning of his last book (J. J. Gibson, 1979/2015, p. 4):

“(…) it is often neglected that the words animal and environment make an inseparable pair. Each term implies the other. No animal could exist without an environment surrounding it. Equally, although not so obvious, an environment implies an animal (or at least an organism) to be surrounded.”

On the one hand, it is an easy assumption that organisms live in an environment. The degree of reciprocity between organism and environment may vary among psychological traditions, but it seems impossible to propose an organism living in total emptiness. On the other hand, the difference between world and environment is key, because the world is the surroundings of the animals described in terms of physics, while the environment is the surroundings described in ecological terms: this is, taking them as related to the organism’s capacities. It is precisely the confusion between environment and physical world what reveals that a dualism is functioning (Michaels and Carello, 1981), implicitly or explicitly. The Gibsonian description of the environment is not a physicalist account of the concept but an ecological one (Lombardo, 1987/2017). This means that physical metrics are not useful to explain behavior as they do not relate to organisms (Richardson et al., 2008). Instead, environmental realities (events, for example, that are related to the organism that perceive them) are the relevant realities for a psychological explanation because they are meaningful: they relate to their capacities. The neogibsonian framework provided a set of good examples of ecological metrics through the mathematical operationalization of affordances, as we will see in section Affordances.

But, how organisms deal with their environments? How can they perceive the affordances -that is, the possibilities for action? Agents need to detect potential damages and take advantage of resources in their environment. Thus, organisms need to detect meaningful information to behave properly. However, sensory stimulation is not sufficient for perception, because there can be sensory stimulation without perception as it happens, for example, in the case of a fog-filled room: light produces the excitement of the specialized photoreceptors, but the agent cannot perceive her surroundings because she cannot see the surfaces of the room (Chemero, 2009, p. 107). However, when the fog is removed, the light reflects differentially in the surfaces of the room, so it constitutes an ambient optic array with the features of the surfaces specified in the structure of the light array. The idea is summarized as follows:

Light originating from an energy source such as the sun is selectively absorbed and reflected by the surfaces of objects (which are the interfaces between the substances of which the objects are made and the medium – in this case, air). As reflected light reverberates off the surfaces, it fills the medium with ambient light. Since the surfaces of objects differ in their orientation to the light source, as well as in shape, texture, pigmentation, and motion, the ambient light takes on a corresponding (heterogeneous) structure (Glotzbach and Heft, 1982, p. 111).

This heterogeneous structure is the ambient optic array, and that is what shows the agent the different possibilities for acting in the environment – hence light is *informative*^2^ about the environment The information in the array is not located in individual points of stimulation, but in the structure of the whole pattern; that is, in higher-order variables. This information is ecological because it shows the way in which the surroundings are disposed in relation to a perceiver’s point of observation. The ecological character is given not only by light itself as a physical energy, but also by the action of the agent. As Gibson claimed, “[a]n affordance, as I said, points two ways, to the environment and to the observer. *So does the information to specify an affordance*” (J. J. Gibson, 1979/2015, p. 132, emphasis added).

Ecological information is informative of the environment because it specifies the available affordances. ‘Specificity’ refers to the idea by which the presence of ecological information corresponds to the direct perception of affordances. Some authors interpret the meaning of specificity as in legal contracts: as a guarantee for the presence of certain elements (for example, a certain pattern of light guarantees a surface) (Käufer and Chemero, 2015, p. 157). Nevertheless, most authors understand specificity as based on natural law. For example, the ambient optic array specifies the environment because of the laws of optics, that is, because each object and surface reflect the light differentially. Specificity, then, can be conceived as the nexus between ecological information and environmental properties.

An important aspect of specificity is that it allows for the lawful character of ecological information. In this sense, “[e]cological information is lawful not in the Newtonian sense of being universal in space and time, but in an ecological sense of being regular within an ecological context of constraint” (Warren, 2005, pp. 342–343). When the agent explores the environment and encounters this ecological information, the agent uses that information for guiding its intentional behavior (Reed, 1983, p. 90). What follows from the detection or picking up is that there is no need to process and enrich the ecological information, since it is sufficiently informative of the affordances of the environment as for guiding the behavior of the agent by itself. In this sense, non-representationalism and the rejection of cognitive processing is a consequence of both the specificity and the picking up of ecological information. For this reason, the detection of ecological information implies the direct perception of affordances.

Ecological psychology focuses on the organism-environment coupling and not on what happens at the subpersonal, neural level. However, this does not mean that this level of description is not important for enabling the whole process of perception. As Travieso and Jacobs (2009, p. 403) claimed: “Note, though, that one cannot reject the validity of studies with smaller or larger levels of analysis; that would be analogous, we think, to rejecting current neurobiology because subatomic particles are not taken into account.” In this sense, J. J. Gibson understood that at a neural level, the systems do not compute, transform, or enrich information, but that they “resonate” to ecological information (J. J. Gibson, 1966, p. 5), an idea that resembles current dynamic approaches to neuroscience (Freeman, 2000; Raja, 2018).

The relation between the ecological approach and cognitive neuroscience can also be seen in the discussion on the two visual streams (ventral and dorsal) proposal by Goodale et al. (1991); Goodale and Milner (1992); and Milner and Goodale (1995), and their linkage to computational and ecological theories, respectively (Norman, 2002). Ecological psychologists question this proposal for two main reasons (Michaels, 2000). The first one was that the so-called ventral system retained all the postulates that sustain the need of inferential and computational process in perception, which, in turn, gave rise to the ecological critique (i.e., the poverty of the stimulus, the passivity of the perceiver, etc.). Second, because the concept of action used to define the dorsal stream was restricted to performatory action, ignoring the exploratory actions in which perception is based according to the ecological approach. In other words, perception for action was described as a unidirectional function, in which perception guided action, not as a perception-action loop.

In addition, it is important to highlight that the “perception for action” functioning of the dorsal stream has been widely accepted by the cognitive neuroscience community. However, there is no computational description of its functioning (the “how”), being their evidences based on behavioral and clinical data. It is assumed, therefore, that the computational-representational level of analysis is not compulsory for describing a psychological function.

### Affordances

‘Affordance’ is the ecological concept that has had a major diffusion since it was coined in the 1960s (J. J. Gibson, 1966, p. 285), although J. J. Gibson used some terms for affordances previously (J. J. Gibson, 1950, pp. 198–199). He proposed ‘affordance’ as an alternative for ‘value’ as it is used in philosophy. During the 1970s, J. J. Gibson (1977) refined the term while his colleagues started to spread it in their works (see, for example, Reed and Jones, 1977; E. J. Gibson et al., 1978). J. J. Gibson (1979/2015) argued that affordances are the main objects of perception in his theory: the possibilities for action that the environment allows for an organism (Reed and Jones, 1982). For example, we do not perceive steps in centimeters, but whether or not we can step on them. Affordances are defined in the organism-environment level of analysis: an organism embedded in her system knows her environment because she perceives affordances.

Gibson claimed that perceiving affordances is perceiving ecological meaning (J. J. Gibson, 1979/2015, pp. 131–132), which is perceiving how the surroundings are related to the agent’s capacities. The idea of affordance shows that an organism does not perceive an objective, value-free, physical world in which meaning is imposed, as in Gestalt theory (see section Gestalt Psychology). We do not create affordances when we perceive them (Michaels, 2000), they already exist in the system as constant relations between organism and environment. The detection of information amounts to affordance perception, so affordances are meaningful objects of perception in an organism-environment system. In Richardson et al. (2008) words:

“[A]ffordances are perceived by detecting lawfully structured information (...) that invariantly specifies features (capabilities) of a particular perceiving-acting agent in relation to features of a particular substance, surface, object, or event. A water surface with adequate tension can afford locomotion for an insect but not a human.” (Richardson et al., 2008, p. 179).

Norman (1988) book originally named *The psychology of everyday things*, later named *The design of everyday things* (1988) is one of the works that popularized affordances. Although E. J. Gibson (2002) did not mention the episode in her biography, Norman (1999) wrote that J. J. Gibson and himself met and discussed the theory of affordances during the spring of 1979, when the Gibsons visited the Salk Institute in California. At the beginning, Norman disliked the theory of affordances, but he defended the importance of the term years later, even discussing the careless use of the concept:

“Far too often I hear graphic designers claim that they have added an affordance to the screen design when they have done nothing of the sort. Usually they mean that some graphical depiction suggests to the user that a certain action is possible. This is not affordance, either real or perceived. Honest, it isn’t. It is a symbolic communication, one that works only if it follows a convention understood by the user.” (Norman, 1999, p. 40).

Despite its centrality within ecological psychology, there is not a single definition shared by all ecological psychologists. We can differentiate between those authors who claim that affordances are properties of the environment that are complemented by aspects of organisms, while there are other authors claiming that affordances are properties of the organism-environment system.

In the first group, Turvey et al. (1981) and Turvey (1992) developed a dispositional theory of affordances, taken as properties of the environment that are complemented by effectivities of animals (causal propensities to effect a particular action, see section Effectivities). Affordances always actualize when both properties are together under the right circumstances (Turvey, 1992, p. 178). Stoffregen (2003) reacts against this view because the idea that affordances are complemented by effectivities implies that affordances are properties of the environment alone, and also thinks that affordances should be better understood as emergent properties of the organism-environment system (Stoffregen, 2003, p. 118). Another aspect of Turvey’s dispositional approach is that, while dispositions never fail to actualize, an animal may fail when is taking advantage of an affordance (Chemero, 2009, p. 145). In this sense, the actualization of an affordance is not as rigid as chemical or physical processes, because they are based on the performance of certain abilities. We think that this emphasis on the physicalist model is the reason behind Reed’s understanding of affordances as aspects of the environment that exert selection pressure (Reed, 1996, pp. 18, 31). Chemero considers that this view is equally problematic, because if animals should conform or adapt to the pressures of affordances, then it breaks the organism-environment mutuality or reciprocity (Chemero, 2009, p. 146). In addition, Chemero (2009, pp. 120–121, 218) considers that affordances should be taken as normative relations of the organism-environment systems, and the set of all affordances for a given organism is its ecological niche. However, this approach is also problematic, since the introduction of normativity in the picture goes against the lawful definition of specificity (as we will see in Specificity) and, therefore, against direct perception.

One of the major advances on the empirical and mathematical study of affordances was made in the famous study of Warren (1984) on climbability. In this study, participants were separated into two groups depending on their height (short people vs. tall people) and they were asked to judge if they could climb on steps of different heights placed in front of them. As expected, tall participants judged as climbable higher steps than short participants. Warren analyzed at which point participants reached a critical step (i.e., a step that, when presented multiple times, in 50% of cases was judged as climbable and 50% of cases as non-climbable). He noticed that the critical step height was related to the body size of participants, and he proposed a biomechanical model that pointed out the importance of body measures: *Rc = Leg + ULeg – Lleg*, being *Rc* the critical step height, *Leg* the leg length, *ULeg* the upper leg length, and *Lleg* the lower leg length. When Warren divided the critical step height (that he empirically found for the two groups of participants) by their leg length, the significant differences between the two groups disappeared. Moreover, he claimed that a person estimates as climbable those steps that are less than 0.88 times her leg length. Thus, this mathematical formulation inaugurated a set of studies that tried to identify the possibilities for action allowed by a surface or object for a given organism in relation to its body measures. His methodology was promptly used for studying other affordances (e.g., Warren and Whang, 1987; van der Meer, 1997).

### Perceptual Learning

Although perceptual learning is an area of research, not a concept or idea, we think it is necessary to delve into this topic to give a more complete view of the foundation and reception of ecological psychology. J. J. Gibson (1966, preface) stated that since 1963 he focused on the development of a theory of perception, while his wife concentrated in perceptual learning. Thus, both branches of the ecological account were developed almost in parallel during the seventies (Rader, 2018) and, therefore, there are subtle differences in their assumptions that might be important for a theory of perceptual learning and development, according to Read and Szokolszky (2018). Before this task division, the Gibsons worked together in the rejection of what they called ‘enrichment theories’ (J. J. Gibson and E. J. Gibson, 1955), as when they claimed: “We have no patience with the attempts to patch up the S-R formula with hypotheses of mediation” (J. J. Gibson, 1967, p. 132). Precisely, Gregory’s theory of indirect perception motivated the unique contribution co-authored by the Gibsons after the task division, due to their common interest in the rejection of enrichment theories (E. J. Gibson and J. J. Gibson, 1972). These theories depart from the poverty and ambiguity of the stimulus and conceive perceptual learning as the process of inferentially disambiguate and enrich the stimulation with previous knowledge. On the other hand, differentiation theories account for a refinement (differentiation) of the stimulation, information, or impressions, and this include Gestalt psychology and the specificity theory (see E. J. Gibson and Pick, 2000, for a more detailed classification of perceptual learning theories and their historical development). The ecological approach to perceptual learning aims to explain how perceivers take advantage of the specific and redundant information available in the ambient energy arrays (E. J. Gibson, 1969; E. J. Gibson and Pick, 2000). This is, perceptual learning has to do with the processes of attunement and calibration. First, organisms must engage into an attunement process (to learn how to detect specific information for affordances); second, they need to adjust their behavior to an informational variable (a calibration process). The most important experimental paradigm for this line of research is known as “the visual cliff,” an experimental procedure developed by E. J. Gibson. It consists on a walkable or crawlable platform that includes an actual cliff covered with a transparent surface, which protects participants from falling, although they still have visual information of the cliff. This was firstly tested on animals, and later on crawling infants. E. J. Gibson and Walk (1960; see also Walk and E. J. Gibson, 1961) showed that, as soon as infants crawled, they perceived a cliff because infants avoid crossing even when their mothers called them from the other side of the cliff. However, it was very difficult to test their behavior before they could crawl (see Rader, 2018, for a recent review on experiments that followed the “visual cliff”).

The reception of E. J. Gibson’s work on development and perceptual learning has been noticeable. Remarkable examples are the work of Adolph et al. (1997) and the direct learning theory detailed in section Development and Learning. In this same vein, a recent proposal reunites the outcomes of J. J. Gibson and E. J. Gibson together with certain developments in evolutionary biology and psychology for establishing a developmental ecological psychology (Read and Szokolszky, 2018).

## Relevant Developments

During the 1970s and 1980s, an increasing number of scholars engaged in the development of the ecological approach. Mace (2015) describes the first expansion of the program in 1970, when Shaw and Lee visited Cornell to study with the Gibsons at the Airport Lab. After this, Lee returned to Edinburgh, where he developed the tau theory described in section Visual Control of Action and Tau Theory, whereas Shaw joined Turvey at the University of Connecticut in 1975. Together with Mace, Carello, Michaels and others they formed what has been named by some authors as the Connecticut School. These scholars had an outstanding role in the foundation of the International Society for Ecological Psychology (ISEP), the journal *Ecological Psychology*, and the Center for the Ecological Study of Perception and Action (CESPA) at the University of Connecticut, the leading center in the deployment of the ecological psychology program. Since then, several developments on ecological psychology were published (Mace, 2015), such as the work on tau theory by Lee, the main publications of the Connecticut school (Turvey et al., 1978, 1981; Michaels and Carello, 1981; Shaw et al., 1982), the work on body-scaled affordances by Warren, the first applications of the ecological theory to social aspects (Heft, 1989; Costall, 1995), the work of Adolph, Eppler, and E. J. Gibson on developmental psychology and learning (Adolph et al., 1993a,b), the first books on J. J. Gibson by Lombardo (1987/2017) and Reed (1988), and the introduction of the metaphor of the polar planimeter by Runeson (1977).

To summarize these developments, first we will resume the studies on visual control of movement and the tau theory developed by Lee, and the work on specificity and effectivities developed by the Connecticut school. We will finish the section by introducing the first developments of several prominent research areas in ecological psychology.

### Visual Control of Action and Tau Theory

Lee started to study the optic flow with the Gibsons (Lee, 2009). After this, he developed his well-known studies with the “moving room” on visual kinesthesis (Lee and Aronson, 1974; Lee and Lishman, 1975), where he showed the strong influence of visual information over non-visual proprioceptive information in the control of stance.

Lee also worked in a mathematical definition of a perceptual invariant capable of explaining the visual control and timing of collisions. He proposed a mathematical invariant specific to time-to-contact, tau (τ), defined as the rate of acceleration of optical expansion (Lee, 1976). Originally, the work focused on collision avoidance, such as braking, but rapidly evolved to preparation for a contact (Lee and Reddish, 1981). In his first study, Lee (1976) used the example of a car following another vehicle that suddenly brakes. How does the driver know how hard should be the brake? Lee realized that distance and speed were not needed for controlling braking and judging the possibility for colliding. Rather, monitoring the expansion in the visual angle of the object in the direction of motion is informative enough to help a driver controlling her action. Through the detection of this rate of expansion, a car following another vehicle can avoid collision thanks to the specification of the “time left until contact at the current speed.”

Tau theory is one of the best-known examples derived from experimental results in the ecological framework, and its applications rapidly expanded (Lee et al., 1983; Craig et al., 2000; see also Lee, 2009 for a review of tau theory). Although the original tau index has been surpassed by later work (Tresilian, 1999), it became a cornerstone that gave rise to an entirely novel approach to the study of the control of locomotion and interception tasks. Researchers have proposed other variables to control braking and movement when an environmental situation affords collision. For example, Zaal and Bootsma (1995) showed that a driver could also adjust deceleration using the rate of change of tau. Other authors have shown that other informational variables in the optic array can affect or be used to estimate the time-to-contact (e.g., Koenderink, 1986; Tresilian, 1999; Michaels et al., 2001).

Undoubtedly, the original work of Lee had a clear influence in the study of the prospective control of movement from a non-representational account, with crucial applications in sports (Fajen et al., 2009), in the description of the information for interception tasks (Michaels and Oudejans, 1992; Bootsma et al., 1997; Fajen and Warren, 2004; Jacobs and Michaels, 2006; Craig et al., 2009), prehension and graspability (Bootsma and van Wieringen, 1992; Newell et al., 1993; van der Kamp et al., 1998); the control of passing through sliding doors (Huet et al., 2009), landing maneuvers in flight simulation (Huet et al., 2011) driving behavior (Land and Lee, 1994), coordination dynamics (Richardson et al., 2016; Akifumi et al., 2017; Nalepka et al., 2017), or human locomotion (Lee and Lishman, 1977; Fajen and Warren, 2003).

### The Connecticut School

Several authors (Cutting, 1982; Chemero, 2009) have pointed to possible differences between the original formulations of J. J. Gibson and the claims of the Connecticut school, mainly because of the strong version of specificity that the latter defended. However, a reconstruction of the historical and philosophical development of ecological psychology, we think, requires the recognition of the position of the Connecticut school both for its epistemological and ontological consistency, and as responsible for the academic settling of the discipline.

In his autobiography (1967), J. J. Gibson recalled his work at the Psychology Unit of the United States air force during WWII, when in the recruitment process they were testing the candidates’ aptitudes for piloting aircrafts, and wrote:

“The so-called ‘spatial’ abilities extracted from existing tests still seem to me unintelligible. The fact is, I now think, that the spatial performances of men and animals are based on stimulus-information of a mathematical order that we did not even dream of in the 1940s (...). And the building of apparatus to simulate the stimulus-information in life situations is difficult when *one does not know what the information is.*” (J. J. Gibson, 1967, p. 136, emphasis added).

Which are the principles proposed by Turvey et al. (1981) for knowing what the information is and how is it lawfully related to the affordances? The concepts of specificity and effectivity are central to explain their position.

#### Specificity

Turvey et al. (1981) proposed a strong version of specificity. That is, the perceptual system detects information that is specific to environmental properties relevant for the organism, the so-called 1:1 relations. There are, of course, redundant and not specific variables to which perceivers may be attuned. But, very much in the pragmatist tradition, those daily actions that are successfully performed, they argued, are based on specific information. The empirical research program of ecological psychology is the description of the ecological information, the specifying variables used in adapted behavior.

Concerning the deployment of the concept of specificity, they proposed a two stages analysis of an affordance: first, to isolate the environmental invariants related to the extension of the affordance; second, to describe the light patterned and to characterize the patterning as optic variables. Thus, by transitivity, the relation between environment and information and the relation between information and affordance allow us to directly perceive the affordance (Turvey et al., 1981, pp. 264–266). This has been characterized as a symmetrical law in which the environment specifies information and information specifies affordances, which leads to a 1:1:1: relation of these elements by specification or unique correspondence (Chemero, 2009, p. 111).

As Turvey et al. (1981), acknowledged, probably the tau theory described in section Affordances constitutes the first empirical example of the accomplishments of this research program. Since this pioneering work of Lee (1976) and Lee and Reddish (1981), several other informational variables of different perception-action systems were described. Among the outstanding ones are the invariants in the inertia tensor in dynamic touch that we will describe in section Other Developments.

#### Effectivities

The Connecticut School centered their efforts in developing the concept of effectivity to answer how behavior can be regular without being regulated (J. J. Gibson, 1979/2015, p. 215). The concept of effectivity (a description of the behavioral capacities of the organism) is necessary for establishing the relevant properties of the environment. Then, a theory of action is needed. Turvey et al. (1978) enunciated the two main problems that such a theory had to face. First, the problem of degrees of freedom; second, the context-variability of motor control.

Previous theories of human movement (e.g., Luria, 1966; Turvey et al., 1978) conceived motor control as the central programming and execution of motor programs that specify each value or degree of freedom of all joints and muscles of the skeletomuscular system. However, as Turvey et al. stated:

“The executive problem in this case is analogous to that of finding the optimum of a function with many variables. Algorithms that theoretically allow the solution of such problem prove to be infeasible in practice” (Turvey et al., 1978, p. 559).

The second problem, context-variability, consists in that motor programs do not guarantee the intended movement. It refers to three main sources of variability: The first one is an anatomical source, by which muscles vary in the role they have in joint movement depending on the anatomical position. The second source of variability comes from mechanical forces, so that the relation of innervation and movement varies if the segment is, for example, already in movement, generating inertial forces. Finally, the third source of variability results from the physiology of the innervation system. That is, the modulation of the motoneurons varies so that the same activation may produce different effects depending on the state of the muscle.

They offered an alternative approach to face these problems by rejecting the idea of the control of individual variables: They proposed that motor control consists of a smaller number of “clusters” or collective variables whose control is internal and relatively autonomous from other clusters. A classic example of these collective variables is the relative phase of the swing of legs and arms when walking (Turvey, 1990). Turvey et al. (1978) proposed an approach consisting in coordinative structures and coalitions. Coordinative structures refer to those collective variables, and they were defined as groups of muscles, often spanning several joints that are constrained to act as a unit. Whereas coalitions refer to the (hierarchical) relations between those structures and the mode of control.

Thus, Ecological psychology turned to the work of Bernstein (1967) on the problems of degrees of freedom (Latash and Turvey, 1996), and to dynamical and complex systems approaches (Kelso et al., 1980; Kugler et al., 1980; Kugler and Turvey, 1987; Beek, 1989; Beek and Turvey, 1992; Kelso, 1995) concerning autonomous control and self-organization. There are, at least, two aspects of dynamical systems that make them a useful tool within ecological psychology. First, a dynamical system is an ensemble of elements forming a unity that changes over time due to the interdependence of the elements that shape the system. Second, the changing behavior of the system is mathematically described and exhibit emergent and self-organized behavior (Richardson and Chemero, 2014). The use of dynamic and complex systems has been especially fruitful in coordination dynamics (see, for example, Stoffregen and Riccio, 1988; Turvey, 1990; Riley et al., 1999).

### Other Developments

Once we have sketched the first developments beyond the work of J. J. Gibson, in this section we try to introduce other areas of research. It is beyond the scope of this paper to give a detailed account of all the work performed in these areas, so we will focus on the studies that initiated and motivated those lines.

#### Dynamic Touch

Contrary to the psychophysical analysis of touch as the sensibility of the skin surface, J. J. Gibson (1962, 1966) defended the active character of the haptic system, including dynamic touch and the prominent role of the exploratory movements of the hands. Thus, the seminal work of Solomon and Turvey (1988) on perception through hand-held objects showed a canonical example of the haptic system conceived as dynamic touching (see section Perception-Action Loop and Perceptual Systems). This work constituted a cornerstone in the study of touch, defining one experimental paradigm and opening a new research field. In his experiments, participants hold a rod from a single grip and had to estimate its length. They could neither see the rods, nor touch their ends. Experimenters found that participants accurately matched the length of the rods. How could they perceive the rod’s length wielding it from a single grip? Authors stated that rotational dynamics played a crucial role. Participants wielded the rod detecting its resistance to rotate (its moment of inertia) through the muscular effort, so they accessed the invariant information present in the inertia tensor of the rod. This tensor is a physical property directly related to the rod’s length that is only accessed when wielding the rods.

Dynamic touch studies rapidly increased the areas of inquiry, including the different movements (torques) needed to diagonalize the inertia tensor (Solomon et al., 1989), how weight is perceived (Amazeen and Turvey, 1996), or the perception of different forms by wielding (Burton et al., 1990; Pagano and Turvey, 1992; Turvey et al., 1992). Moreover, several studies proposed that even proprioception (in particular, the position of the extremities) is specified through their inertia tensor (Pagano and Turvey, 1992; Pagano et al., 1996). A detailed review on rotational dynamics and dynamic touch has been written by Carello and Turvey (2000).

#### Affordances

The mathematical operationalization of affordances started with the classic work of Warren (1984) on the perception of climbability (reviewed in section Affordances) and continued with the perception of passability (Warren and Whang, 1987). This methodology has been applied to the study of several affordances. According to Fajen et al. (2009), affordances can be divided in two groups: body-scaled affordances and action-scaled affordances. The first group refers to properties related to the body dimensions of organisms. This is because the goal-directed action needs specific dimensions of the environment to fit the action; for example, the grasping pattern of an object depends of the relation of object and hand sizes (e.g., Newell et al., 1993; Cesari and Newell, 2002). The second group of affordances are those properties related to the action-capabilities of the organism. That is, the affordances are environmental properties that allow action in relation to dynamic properties of the perceiver’s movement. Classic examples of this type are optic variables that specify the point of interception of a flying ball that is visually tracked by a moving observer (e.g., Oudejans et al., 1996).

After the publication of J. J. Gibson’s seminal work (J. J. Gibson, 1979/2015) the term has been continuously spreading among a wide variety of academic contexts, from architecture (Maier et al., 2009; Rietveld and Rietveld, 2017) to robotics and artificial intelligence (Effken and Shaw, 1992; Duchon et al., 1998; Chemero and Turvey, 2007; Rome and Dorffner, 2008). As an example, a Google Scholar’s search^3^ for the term in the decade of 1980–1989 yielded 593 results, while in the current decade (2010–2018) yielded 23500 results. The most important problem related to this impressive growing is the variety of meanings for the same word (Oliver, 2005). For example, several authors argued that affordances are some kind of mental representations (see for example, Vera and Simon, 1993; Millikan, 2005), which contradicts direct perception and the reciprocity of organism and environment. Important discussions on the concept of affordances within ecological psychology can be found in Chemero (2003), Michaels (2003), and Stoffregen (2003). Reviews on the application of the concept of affordance can be found in different areas: learning and development studies (Adolph et al., 1997), visual control of action (Fajen, 2007) and sports (Fajen et al., 2009).

#### Development and Learning

As we could see in section Perceptual Learning, E. J. Gibson defined development and learning as the process of using available information to adjust behavior. The work of Adolph followed this path studying how infants developed the perception of affordances for locomotion (Adolph et al., 1993a,b). Afterward, she extended her work to the study of the transition from crawling to walking. Adolph studied crawling longitudinally and systematically, describing the crawling behavior of infants over different slopes. Adolph showed that infants engaged in a circular behavior in which improvements in crawling produced better estimations of slope inclination, and, conversely, better estimation produced improvements in the crawling behavior. Furthermore, Adolph et al. (1997, 1998) stated that no transfer of the slope estimations happens in the transition from crawling to walking, so infants had to learn new attunements.

One of the latest developments in perceptual learning is the theory of direct learning (Jacobs and Michaels, 2007). This theory defines a perceptual task in an informational space. In such space, each point represents an informational variable and trajectories from point to point represent the learning process. The innovation of this approach lies in the mathematical apparatus that let us observe the direct character of learning represented by a path in the informational space. This path is constrained by the vector field which represents the discrepancies between judgments (actions) and feedback (outcomes). In other words, learning depends on the convergent information that (directly) pushes the agent to move from non-specific invariants to (more) specific invariants, and this explains improvements in performance. Thus, this theory states a direct learning hypothesis (e.g., Jacobs et al., 2009).

This approach has been successfully applied to describe perceptual learning in the form of change in variable use (Runeson et al., 2000; Jacobs et al., 2001), and in calibration (Bingham et al., 2000; Jacobs and Michaels, 2006). And it has been applied also to several perceptual tasks, like touch (Michaels et al., 2008; Withagen and Van Wermeskerken, 2009), or landing maneuvers in flight simulators (Huet et al., 2011).

#### Social Coordination

In section Effectivities we saw that ecological psychology approaches the problem of motor control through collective variables. A classic example of this is the coordinated swing of arms and legs, a field of study called coordination dynamics. Schmidt et al. (1990) showed that the patterns of interlimb coordination of the lower legs oscillations between two people produce similar coordination patterns to that of the two legs of a person, showing that the latter is not a pure mechanical event. This study started a research area relating coordination dynamics and social behavior called social coordination. Since the publications of these results, studies on social coordination has been an increasing field of research from an ecological dynamic framework (see Schmidt and Richardson, 2008; Marsh et al., 2009).

#### Sports

One of the related areas of applied research that has been greatly influenced by the concepts and research framework of ecological psychology is sports. Some of the most relevant studies in this field made use of outcomes achieved in the study of timing and interception tasks and applied it to ball sports (e.g., Bootsma and van Wieringen, 1990; Peper et al., 1994; Craig et al., 2000; Zaal and Michaels, 2003). Nonetheless, research on sports was not limited to the analysis of ecological information, but also to the dynamics of actions in sports (Araújo et al., 2006). Representative publications summarizing research results and theoretical position on this area can be found in Araújo and Davids (2009), Beek (2009), and Fajen et al. (2009).

## Conclusion

How is ecological psychology related to current approaches to perception and action? Is neuroscience completely irrelevant to ecological psychology? We think that ecological psychology can be complemented by developments in other areas, as well as other approaches can benefit from the results of ecological psychology. For example, some approaches and disciplines are currently accommodating an analysis of affordances, a concept that has gained popularity in the recent years beyond the scope of ecological psychology. This object of study and the way it is analyzed by ecological psychology may illuminate the complementarity of different approaches in order to offer a wider picture of how psychological processes develop. For example, a subpersonal analysis of neural processes in the brain can be complemented by an upper-level analysis of which behavioral patterns are used by the organism to pick-up ecological variables in the environment. In this sense, neuroscience is not completely irrelevant to ecological psychology: an analysis of the brain based on neural dynamics complements the ecological approach since it offers an explanation of how ‘resonance’ works (Raja, 2018), as it was suggested by J. J. Gibson (1966, p. 5). In this sense, as the influence of ecological psychology grows beyond its own field of study, this very discipline can benefit from the outputs of other approaches, specially the ones related to neurodynamics (Freeman, 2000).

In this paper, we have presented a sketch of the theoretical foundations and the historical development of ecological psychology as a discipline, as well as its main innovations and applied contributions for the philosophy of mind and the cognitive sciences. We have tried to show that ecological psychology offers one of the most powerful alternatives for developing a non-representational and non-dualistic psychology because of two reasons. First, because of the radical philosophical position developed mainly by J. J. Gibson that confronted the main weaknesses of the psychological theory, which are the organism-environment, perception-action and objective-subjective dichotomies. The second reason is that an increasing number of scholars have engaged in the ecological program, providing a growing empirical corpus. We hope that this paper helps illustrating the theoretical innovations and methodological contributions of ecological psychology.

## Author Contributions

All authors have made an equally substantial contribution to the work.

## Conflict of Interest Statement

The authors declare that the research was conducted in the absence of any commercial or financial relationships that could be construed as a potential conflict of interest.

## Footnotes

1 As J. J. Gibson (1967/1982) stated, the person that most influenced his views was his wife, E. J. Gibson, who made similar claims about her husband in her autobiography (2002). However, here we focus exclusively in the theories that influenced Ecological Psychology.

2 Marr’s approach to vision differs substantially from J. J. Gibson’s, since the former proposed an information-processing view that was rejected by the latter. Marr (1982/2010, pp. 29–31) analyzed the ecological approach and claimed that J. J. Gibson did a great job in answering how one can obtain constant perception on the basis of continually changing stimulation. Nevertheless, Marr rejected the ecological approach since he seriously underestimated the complexity of the information-processing problems in vision. The problem is that J. J. Gibson himself claimed that ecological information was not the kind of information as found in the information-processing approach, since he considered that this latter kind of information was based on Shannon’s mathematical theory of communication, and it implied a sender, a receiver, and a signal that transmitted a message that had to be reconstructed. This kind of information for communication is not the same as the one found in the ambient optic array. For this reason, “[t]he information that can be extracted from ambient light is not the kind of information that is transmitted over a channel. There is no sender outside the head and no receiver inside the head” (J. J. Gibson, 1979/2015, p. 57).

3 Search performed on May 23, 2018.
